# Effect of Sonication on the Properties of Flaxseed Gum Films Incorporated with Carvacrol

**DOI:** 10.3390/ijms21051637

**Published:** 2020-02-28

**Authors:** Shiyuan Fang, Weiqiang Qiu, Jun Mei, Jing Xie

**Affiliations:** 1 College of Food Science and Technology, Shanghai Ocean University, Shanghai 201306, China; b15850777@163.com (S.F.); wqqiu@shou.edu.cn (W.Q.); 2 National Experimental Teaching Demonstration Center for Food Science and Engineering Shanghai Ocean University, Shanghai 201306, China; 3 Shanghai Engineering Research Center of Aquatic Product Processing and Preservation, Shanghai 201306, China; 4 Shanghai Professional Technology Service Platform on Cold Chain Equipment Performance and Energy Saving Evaluation, Shanghai 201306, China

**Keywords:** sonication, active films, flaxseed gum, antibacterial agent, carvacrol

## Abstract

Carvacrol is a natural compound known to be a highly effective antibacterial; however, it is a hydrophobic molecule, which is a limitation to its use within food packaging. Flaxseed gum (FG) films containing different contents of carvacrol (C) were produced by a film-casting method with sonication. The effects of sonication power and time on the properties of the FG-C films were investigated by measuring the film thickness, mechanical properties, contact angle, opacity, water vapor permeability (WVP), water sorption isotherm, Fourier transform infrared spectroscopy(FTIR), differential scanning calorimetry (DSC), antibacterial and antioxidant activities, and microstructure. The results showed that sonication power and time had significant effects on mechanical and barrier properties, film opacity, and degradability (*p* < 0.05). The tensile strength (TS) and elongation at break (EB) values exhibited an obvious improvement after sonication, and FG-0.5C-6030 had the lowest TS (33.40 MPa) and EB (4.46%) values. FG-C films formed a denser structure and the contact angle was improved as a result of sonication, which improved the integration of carvacrol into the FG matrix. In terms of microstructure, sonication resulted in a homogeneous and continuous crosssection of FG-C films, and regular surface and cross-sectional images were obtained through the highest acoustic intensity and longest time treatment. The FG films incorporated with carvacrol displayed antibacterial properties against *Staphylococcus aureus*, *Vibrio parahaemolyticus*, *Shewanella putrefaciens*, and *Pseudomonas fluorescens*, as well as increased antioxidant properties, and sonication was proven to enhance both of them.

## 1. Introduction

When directly applying antibacterial agents onto a food surface, their antibacterial activities are reduced owing to their quick spread into foods, denaturation with the food components, and inhibition of food-borne pathogens. Antibacterial agents in edible films can slowly and continuously migrate from the packaging material to the surface of the food and extend the shelf life of food. In recent years, many edible film materials have been studied, including polysaccharides, proteins, and lipids [1]. Flaxseed gum (FG) is a heteropolysaccharide and has been found to be suitable as an emulsifier, thickener, and stabilizer in the food industry [2,3,4]. The functional properties of FG are equivalent to those of Arabic gum [5] and could be used to enhance the emulsion stability by improving the viscosity and weakening the interfacial tension [6]. FG combined with lemon grass essential oil had a good effect on the preservation of ready-to-eat pomegranate arils [7]. Carvacrol is generally recognized as safe (GRAS) by the U.S. Food and Drug Administration (USFDA), and has been applied to improve the safety and freshness of food on account of its broad-spectrum antibacterial activity [8]. Alves et al. [9] found that chitosan films with grape seed extract and carvacrol microcapsules could increase the shelf life of refrigerated salmon to 4–7 days of storage owing to the antibacterial effect of the natural agents. However, when adding antibacterial agents to edible films, it is difficult to mix them evenly, while ultrasonic technology, as a physical method, can enhance the force between film molecules to form a dense film structure [10]. Sonication is propagated via a series of compression and rarefaction cycles induced by sound waves to the molecules of medium. When the sonication power is high enough, the rarefaction may exceed the attraction among molecules in the medium, resulting in the formation of cavitation bubbles. A huge number of cavitation bubbles are formed, grown, oscillated, and burst during the propagation of ultrasound, and high local pressure (5 GPa) generates in a very short time, which causes the emulsion droplets to be dispersed uniformly. Borah et al. [11] prepared potato peel and sweet lime films with sonication, and reported that a film with better properties could be obtained by increasing the sonication time. Rodrigues et al. [12] reported that sonication caused mesquite seed gum and palm fruit oil emulsion droplets to be dispersed more uniformly, with their surface hydrophilicity and tensile strength (TS) increased.

As far as we know, there are some studies about active films produced from FG [3]. However, there is no research about sonication of FG films. In order to promote the mixing of carvacrol and FG, sonication was employed, and the interactions between these components were analyzed.

## 2. Results and Discussion

### 2.1. Thickness

The preparation of FG films incorporated with carvacrol (FG-C) was carried out by a solution casting method, and samples arelisted in Table 1. The film thickness only changed slightly from 0.080 to 0.085 mm, and thus remained relatively stable (Table 2). This result suggests that sonication did not affect the thickness variation of the prepared FG-C films. FG, flaxseed gum; C, carvacrol.

### 2.2. Tensile Strength (TS) and Elongation at Break (EB)Results

TS represents the resistance to tension force, and EB reflects the film stretching capacity. They are the parameters closely related to the mechanical properties and chemical structure of active films [13]. The incorporation of carvacrol and sonication had significant effects on TS and EB (*p <* 0.05, Table 2). With the increasing content of carvacrol, the TS of FG-C film decreased gradually, but the EB slightly increased, which is in agreement with the findings of Klangmuang and Sothornvit [14] and Gul et al. [15]. When the carvacrol content was the highest (2.0 mL/L), the TS was the lowest. The decrease in the TS of the FG-C films was mainly owing to the addition of carvacrol, which could partially replace the interaction between the FG molecular chains [16]. On the other hand, FG-C films with sonication increased in TS and EB significantly (*p* < 0.05) compared with untreated samples. The increase in sonication power and time led to an increase in the TS and EB of the FG-C films. Some researchers have shown that sonication can make the carvacrol droplets in the film-forming solution smaller, thereby enhancing the interaction between molecules [17]. Compared with other samples treated with sonication, FG-2.0C-4015 (15 min, 400 W) displayed the lowest TS (21.81 MPa) and EB (2.66%) values. This phenomenon could be highly relevant to the application of sonication and the content of carvacrol, as FG-4015 (15 min, 400 W) had the lowest power and highest content of carvacrol among the sonication-treated samples. Excessive sonication caused the film temperature to rise, broke the molecular chains, and destroyed the structure of the network. The longer sonication time and the greater power led to higher TS and EB, which may be achieved by maintaining a more homogeneous dispersion of carvacrol particles in the FG matrix [12]. Pérez-Gago and Krochta [18] reported that the TS increased as the particle size decreased, possibly owing to increased film matrix immobilization at the interface of carvacrol particles. Similar results were also found inmethylcellulose and mesquite seed gum films [12,19].

### 2.3. Barrier Properties against Water via Water Vapor Permeability (WVP) Tests

The water resistance capacity of the FG-C films was evaluated by *WVP*. The adsorption and penetration of water vapor is especially important in food packaging and preservation [20]. Carvacrol could effectively block the transport of water molecules and significantly (*p <* 0.05) decrease the *WVP* owing to its hydrophobic characteristic (Table 2). When carvacrol content increased from 0.5% to 2.0%, the *WVP* increased markedly from 1.90 to 2.51 × 10^−11^g H_2_Om^−^^2^s^−1^MPa^−1^ (*p* < 0.05). It has been demonstrated that water vapor transfer generally occurs through the hydrophilic portion of the film and depends on the hydrophilic/hydrophobic ratio of the film components [21]. On the one hand, the binding of water molecules to the films and the penetration into the interior of the films are prevented because of the presence of carvacrol, improving the moisture barrier properties of the films [22]. The homogeneity and integrity of FG films were poor with carvacrol addition, which may overcome the barrier effect produced by carvacrol. Similar results were observed when the concentrations of thyme essential oil were increased in quince seed mucilage films [23] and gelatin films [24], respectively. Carvacrol is the main active component in thyme essential oil. However, some researchers have reported that the incorporation of essential oils can reduce the WVP of polysaccharide-based active films [25], which is governed by the increasing proportion of hydrophobic components [22].

Sonication had a significant effect (*p* < 0.05) on the *WVP* values of FG-C films. The lowest *WVP* value (1.10 × 10^−11^g H_2_Om^−^^2^s^−1^MPa^−1^) was obtained in FG-0.5C-4030 (30 min, 400 W), approximately 55% lower than that of FG-0.5C-6015 (2.74 × 10^−11^g H_2_Om^−^^2^s^−1^MPa^−1^, 15 min, 600 W). For polysaccharide-lipid blending films, the microstructure depended on the size and shape of lipid droplets in film-forming solutions and their development during film formation [26], which has a great influence on the moisture resistance. During the preparation of film-forming solutions, high sonication power produced a cloud of bubbles in the solutions and these bubbles grew to a critical size and ruptured violently. The entire process of bubble formation, growth, and rupture in a sonication field is called sonication acoustic cavitation [27], which generates extreme temperature and pressure within the collapsing bubbles. Under such conditions, carvacrol droplets were broken down into smaller particles. Subsequently, the carvacrol particles became more homogeneous in the FG matrix and formed an efficient barrier against moisture transfer. However, the extending of sonication time by 15 min resulted in an increasing of the *WVP* values of FG-C films. The *WVP* of FG-0.5C-4015 (2.72 × 10^−11^g H_2_Om^−^^2^s^−1^MPa^−1^, 15 min, 400 W) presented a 43 % increase compared with FG-0.5C (1.90 × 10^−11^g H_2_Om^−^^2^s^−1^MPa^−1^). Previous studies have shown that the more compact the polymer networks in the blending films caused by cross linking, the lower the *WVP* value [28]. The *WVP* of FG-0.5C-4030 (1.10 × 10^−11^g H_2_Om^−^^2^s^−1^MPa^−1^, 30 min, 400 W) presented a 72% decrease compared with FG-0.5C. Owing to the extreme temperature and pressure generated by the collapse of bubbles, there might be a large amount of space in the FG matrix, providing channels for the transfer of water vapor molecules. These results are in line with a previous research study on the effect of sonication on the *WVP* of soybean protein isolate-based film containing lipids [29].

**Figure 1:**
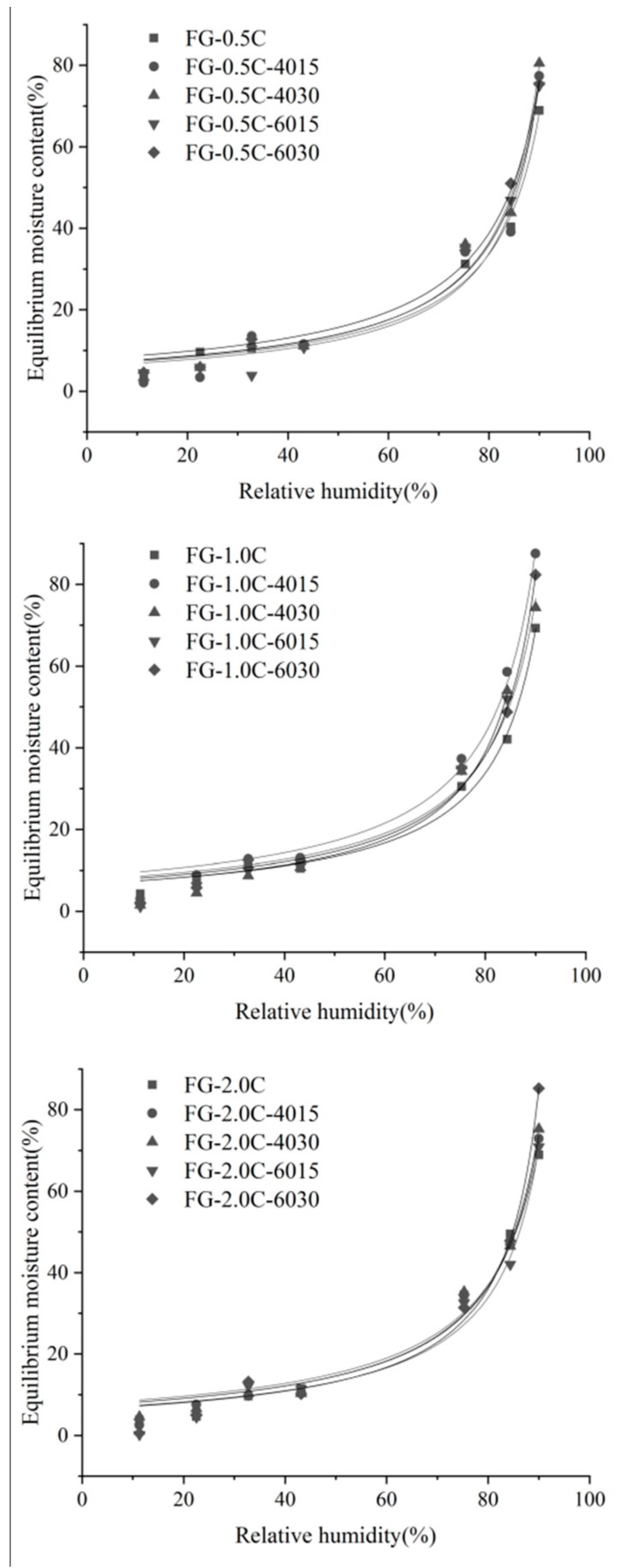
Fitting curves of isothermal hygroscopic Guggenheim–Anderson–de Boer (GAB) model of flaxseed gum (FG)-carvacrol (C) films with ultrasonic assisted treatment.

Guggenheim–Anderson–de Boer (GAB) model parameters of different treatments were calculated and are shown in Table 3. These results indicated that sonication and the incorporation of carvacrol had significant effects (*p* < 0.05) on the water adsorption of FG-C films in the whole range of a_w_ (0.11–0.90) studied. As shown in Figure 1, the water absorption of the FG-C films was low and then increased exponentially when the *a_w_* value was 0.80. Incorporation of carvacrol reduced the number of the membrane surface active sites that can be used for water molecule interaction. Sonication-treated FG-C films could improve the combination of carvacrol droplets and the FG matrix to form a more compact structure, which provided a good barrier performance for the film. The more water molecule adsorption points and the stronger the water vapor adsorption capacity, the higher the value of *W_0_*. The *C* value (constant of the model) is related to the adsorption enthalpy and represents the affinity between water molecules and the membrane surface. A higher *C* value represents the stronger the binding force of water molecules in the monolayer to the adsorption site and the greater the enthalpy difference between the monolayer and the multilayer molecules. The results show the same trend as the single layer moisture content (*W_0_*) according to the moisture content of each type of film. All *k* values (constant of the model) are less than 1, indicating that the model selection is correct. The *k* value of the film samples is relatively constant and the interaction intensity between the water molecule and the film adsorption points are similar.

### 2.4. Surface Hydrophobicity via Contact Angle Measurements

**Figure 2:**
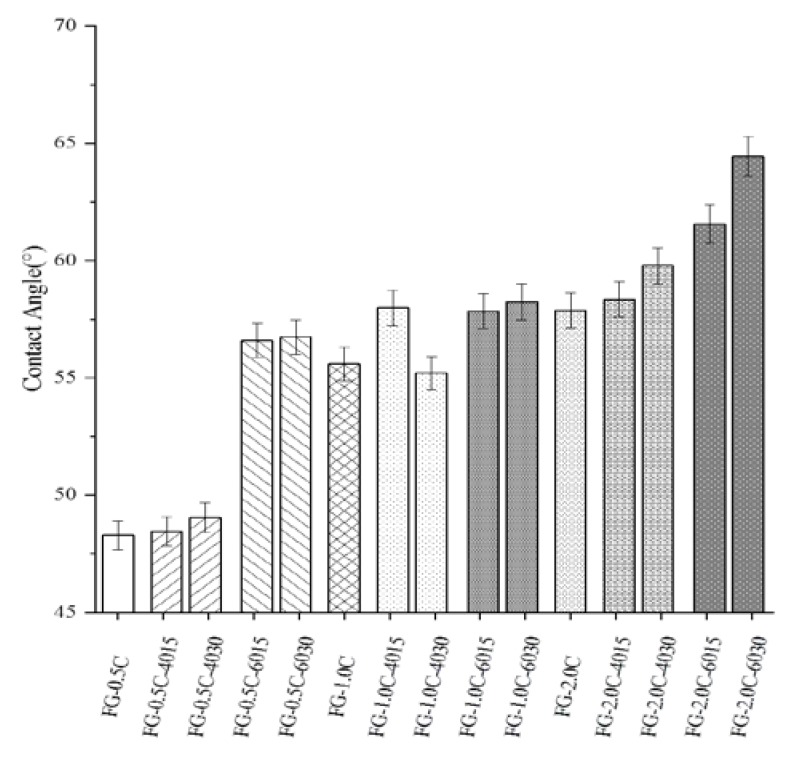
Effect of sonication on contact angle results of flaxseed gum films incorporated with carvacrol (The bars with the same pattern had the same carvacrol concentration and sonication power).

The contact angle measurement is one of the basic wetting properties of packaging materials and an index of the hydrophilic or hydrophobic properties of materials [30]. FG-0.5C film showed a contact angle of 46° (Figure 2), indicating the hydrophilic nature of the FG film. The hydrophobicity of the surface of the FG film was remarkably improved, and thus the contact angle increased remarkably with the increased carvacrol content. The surface possessed a hydrophobic character if the contact angle was larger than 90° [31]. Increased carvacrol content in the FG-C films could reduce the intermolecular attraction within the dense network structure, which caused water droplets to penetrate faster. Carvacrol is hydrophobic, so the contact angle increased with the increase in the content of carvacrol [32]. Increased sonication power and time could increase the possibility of microbubbles in films produced by the sonication [32]. Therefore, sonication could promote the combination of carvacrol and the FG matrix to form a denser structure, thus increasing the contact angle of FG films.

### 2.5. Film Opacity

The opacity of film is vastly influenced by carvacrol addition and the internal structure of films (see Table 4). The internal structure of FG-C films is greatly influenced by the size of carvacrol droplets, the volume fraction of the dispersed carvacrol, coalescence, and emulsification during the drying process [15]. As listed in Table 2, the opacity of FG-0.5C, FG-1.0C, and FG-2.0C is 0.925, 0.866, and 0.821 mm^−1^, respectively. The decrease was the result of carvacrol droplets distributed throughout the FG matrix to promote light scattering [33,34]. Sonication significantly (*p* < 0.05) reduced the film opacity owing to the decrease in the size of carvacrol droplets in the FG-C film-forming solutions, and the rough surface of non-sonication FG-C films decreased the opacity for the light scattering effect. Nevertheless, it is worthy to note that the presence of sonication had a remarkable effect in increasing film opacity, as the sonication FG-C films appeared to be transparent, thus indicating that the sonication allowed for obtaining more homogenous carvacrol droplets inside the FG film matrix, as confirmed by scanning electron microscope (SEM) experiments (see below).

### 2.6. Film Thermal Stability via Differential Scanning Calorimetry (DSC) Analysis

**Figure 3:**
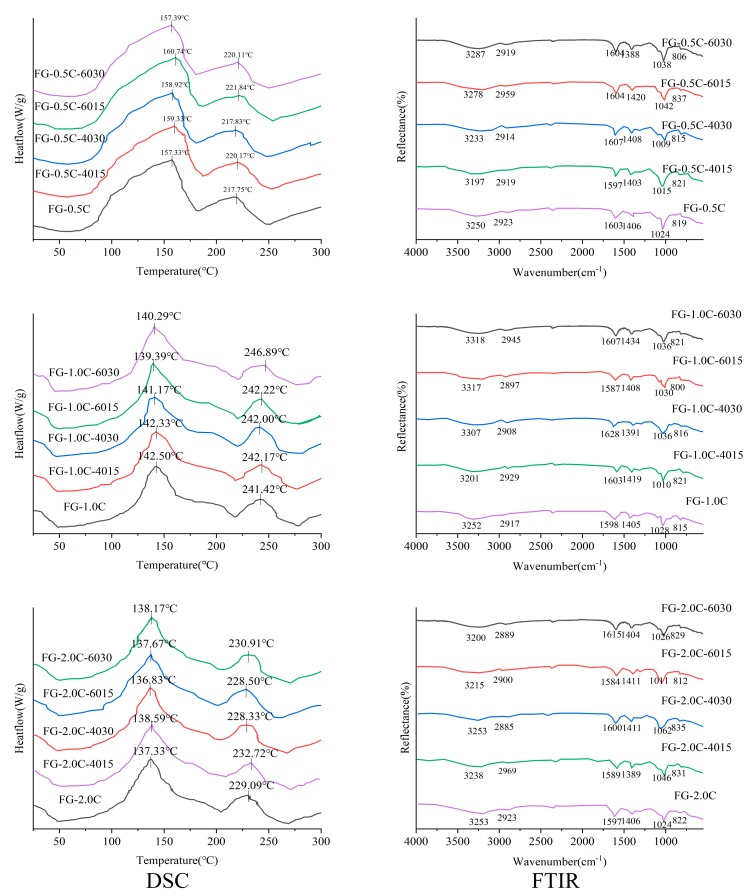
Effect of sonication on differential scanning calorimetry (DSC) (left) and Fourier transform infrared spectroscopy (FTIR) (right) results of flaxseed gum films incorporated with carvacrol.

It is necessary to measure the intermolecular structural changes caused by temperature changes to determine the thermal resistance of FG-C films. The interaction between FG and carvacrol was judged by the changes in the peak shape and appearance temperature in DSC analysis. As shown in Figure 3, DSC curves exhibit the thermally induced endothermic changes of FG-C films between 25 and 300 °C. The thermal properties of FG-C films depend mainly on their chemical compositions and state transitions that occur during drying processing [2]. Heat flow changes in thermal transitions occurring between 80 and 100 °C were mainly related to water evaporation associated to the hydrophilic groups in the FG structure [35,36]. Subsequently, a decomposition step observed at about 230–240 °C was linked with decomposition and depolymerization of FG-C films. Figure 3 shows the DSC thermograms for FG-C films with/without sonication. FG-C films had only one crystallization temperature, indicating that FG had good compatibility with carvacrol. FG-0.5C presentedan endothermic peak at 157.33 °C, and a higher carvacrol content presented a lower endothermic peak temperature. The addition of carvacrol had a significant (*p* < 0.05) effect on the endothermic peaks; however, some of the evaporation-related thermal transitions shifted to higher temperatures with the inclusion of carvacrol. The DSC degradation peaks for FG films incorporated with 0.5%, 1%, and 2% carvacrol appeared at 217.75, 241.42, and 229.09 °C, respectively, which showed that an adequate content of carvacrol slightly improved the thermal stability of FG films. The decrease in thermal stability may be because of higher carvacrol contents loosening the molecular structure of the films. This outcome is consistent with the results obtained by Gursoy et al. [37] and Thi et al. [38]. They observed that adequate contents of essential oils in chitosan and karaya gum-based films significantly improved their thermal stability. The DSC degradation peaks for FG-C films with sonication appeared at higher temperatures related to evaporation, which indicated that the thermal stability of FG-C films was slightly improved by sonication.

### 2.7. Fourier Transform Infrared Spectroscopy (FTIR) Spectra

The FTIR spectra of FG-C films are shown in Figure 3. In the spectra, a wide peak at 3312 cm^−1^ indicates the stretching vibration of free, as well as inter-and intra-molecular, OH groups. The peaks at 2922 cm^−1^ and 1410 cm^−1^ were attributed to the vibration stretching containing CH_2_ and C–OH groups, respectively. The asymmetric stretching vibration absorbance at 1612 cm^−1^ was attributed to the coupling of C–O. The peak at 1410 cm^−1^ was the absorption band of C–OH. The sharp peak at 1050 cm^−1^ was attributed to the coupling of C–O (in a pyranose ring), C=C stretching, and C–OH bending, respectively [39,40]. As for the infrared spectra of FG-C films, the wave number ranges of 2900–3500 cm^−1^ and 1000–1100 cm^−1^ were relatively sensitive to the changes in composition of these films. After the addition of carvacrol, the O–H absorption peaks shifted from 3253 cm^−1^ in FG-2.0C to 3200 cm^−1^ in FG-2.0C-6030 (30 min, 600 W), which obviously moved to a lower wave number owing to the binding interactions between FG and carvacrol. The changes of the peaks at 1612 cm^−1^ and 1410 cm^−1^ were also because of the strong interaction between FG and carvacrol. At the same time, the intensity of the O–H absorption peak decreased and hydrogen bonds between film and water decreased, which indicated that the hydrophilicity of the FG-C films was weakened. This is because carvacrol is a hydroxyl-containing hydrophobic substance interacting with macromolecular components in FG films to form hydrogen bonds through surface hydroxyl groups. The addition of carvacrol increased the hydrophobic groups in the films, which strengthened the vibration absorption peak at 2922 cm^−1^. The cavitation effect of the sonication wave could destroy the hydrogen bonds formed between FG and carvacrol to free the intermolecular hydroxyl groups and increase their absorption peak intensities. In addition, the super-mixing effect enhancing the motion of molecules could accelerate the formation and fragmentation of hydrogen bonds [41].

### 2.8. Film Morphology via SEM Observations

**Figure 4:**
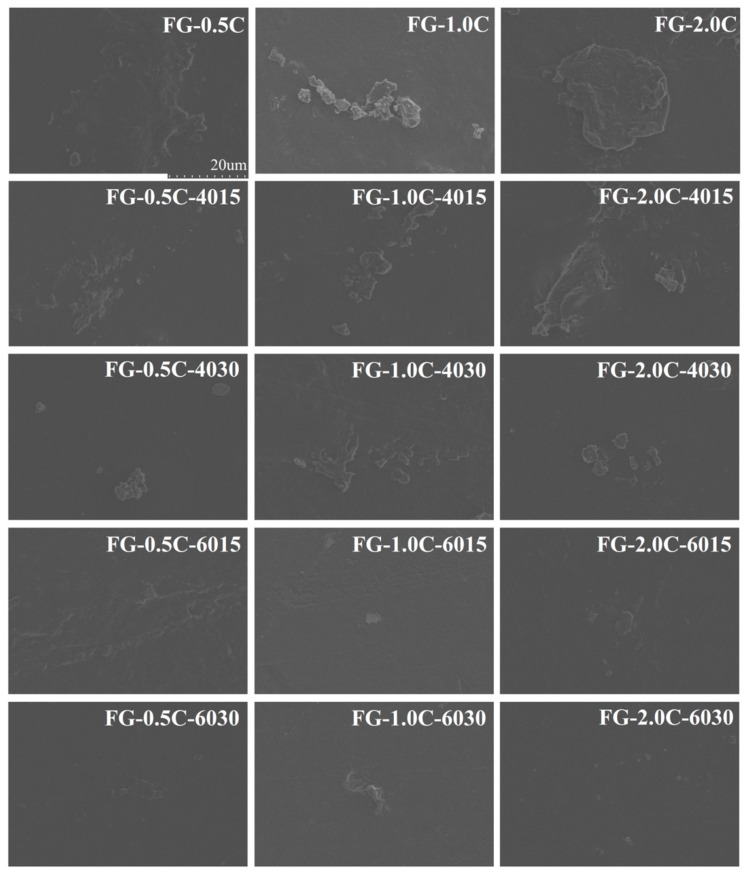
Effect of sonication on scanning electron microscope (SEM) micrographs (magnification: 2000×) of surfaces of flaxseed gum films incorporated with carvacrol.

**Figure 5:**
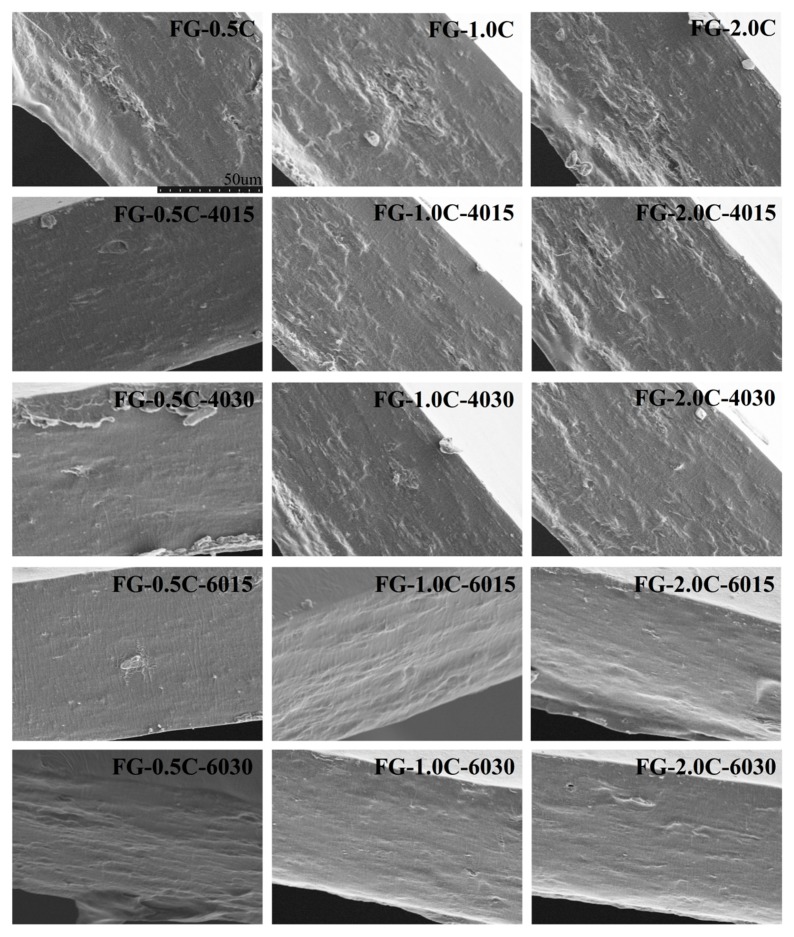
Effect of sonication on SEM micrographs (magnification: 2000×) of crosssections of flaxseed gum films incorporated with carvacrol.

FG-C films with/without sonication were rather smooth and homogeneous (Figure 4). However, the cross-sectional morphologies of the films are different (Figure 5). Similar results have been reported by Baek et al. [42] in tara gum films containing oleic acid and by Kaya et al. [43] in chitosan films containing *Berberis crataegina* seed oil, respectively. The FG-C films formed a homogeneous structure without pores, indicating that the FG-C film-forming solution had a stable emulsion system and remained stable during the drying process [44]. 

In FG-C films without sonication, the continuity of the crosssections of FG films was destroyed owing to the presence of carvacrol (Figure 5). Many white substances could be observed in the SEM images of the control group. With the treatment of sonication, the white substances became smaller, less, and shallower, and after 30 minutes of sonication, most of the white matter substances. However, in sonication-treated samples, the microstructures showed more continuous trends with increased sonication power andtime. FG-C-6030, prepared under the condition of 600W and 30min, exhibited a homogeneous phase with the smallest size of discontinuities in the FG matrix. Similar results have been obtained in methylcellulose-based films containing stearic acid prepared with sonication, and the enhancement of sonication resulted in a more homogeneous distribution of the methylcellulose/stearic acid blending films [45]. Therefore, sonication provided a similar effect on the homogenization by decreasing carvacrol particles and inhibiting carvacrol self-association. Marcuzzo et al. [46] reported that sonication improved the appearance of the final film, and the number and size of polymer aggregates were decreased. After the addition of carvacrol, anamorphous structure was formed in the crosssections of FG-C films with vacuoles and cavities distributed. The volatilization of carvacrol in the drying process will leave some holes, leading to the destruction of the homogeneity and compact structure of the film [15,47]. FG-C films with sonication improved the integration of carvacrol droplets into the FG matrix, resulting in a denser structure that provided good barrier properties to the film. The cross-section image of FG-C films produced without sonication showed a discontinuous microstructure. However, sonication of FG-C films reduced the discontinuity of the crosssection of FG-C films, and homogeneous cross-sectional and smooth surface images were obtained by the higher power and longer time (FG-6030) treatment.

### 2.9. Antibacterial Activity

**Figure 6:**
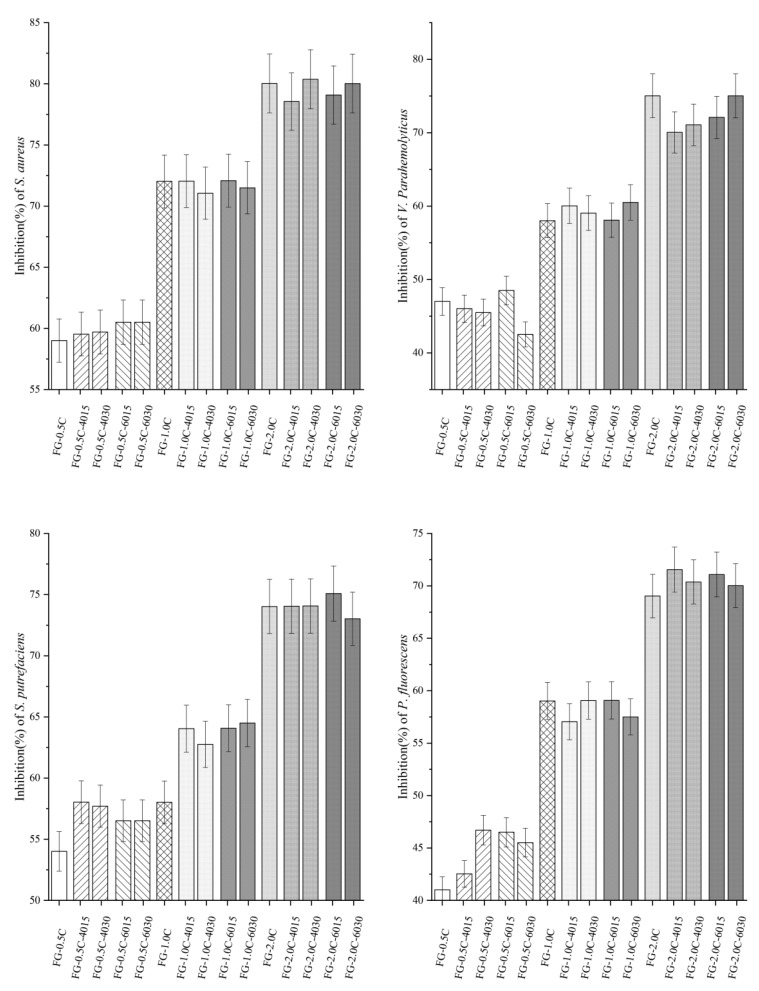
Effect of sonicationon antibacterial activities of flaxseed gum films incorporated with carvacrolagainst *Staphylococcus aureus*, *Vibrio parahaemolyticus*, *Shewanellaputrefaciens*, and *Pseudomonas fluorescens* (The bars with the same pattern had the same carvacrol concentration and sonication power).

Carvacrol is the major component of oregano and thyme essential oil [48]. Many studies have shown that the antibacterial effect of carvacrol against Gram-positive bacteria is stronger than that against Gram-negative bacteria, which may be because of the fact that the cell wall of Gram-negative bacteria is covered with a membrane containing lipopolysaccharide, limiting the diffusion of hydrophobic compounds through the membrane surface [49,50]. Antibacterial activity of FG-C films with/without sonication against Gram-positive (*Staphylococcus aureus*) and Gram-negative (*Vibrio parahaemolyticus*, *Shewanella putrefaciens*, and *Pseudomonas fluorescens*) bacteria was determined by an agar diffusion method, and the results are expressed as an inhibition ratio (%) in Figure 6. All tested FG-C films showed satisfactory antibacterial activity against the tested strains. As expected, FG films with higher carvacrol content increased in antibacterial activity. Sonication of film-forming solutions at different powers and time could significantly increase the antibacterial activity of FG-C films (*p* < 0.05), which could be mainly because of the increase of sonication power and time improved sonication intensity in the current range. As the sonication intensity increased, the distribution of carvacrol became more even, and it could be better released into the solution, and more carvacrol molecules were exposed to the microorganism membrane. In addition, the effect of the releasing rate of the carvacrol could partly explain this phenomenon.

### 2.10. Antioxidant Activity

**Figure 7:**
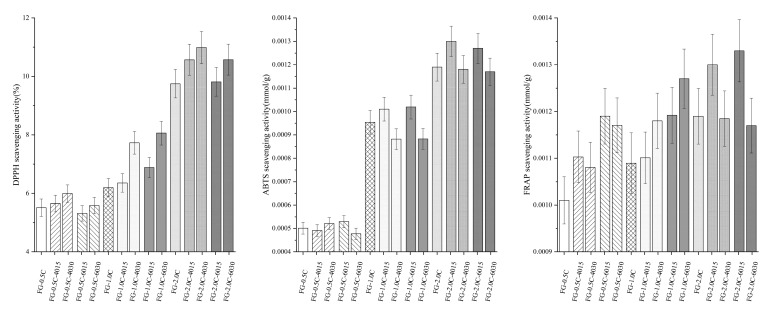
Effect of sonication on antioxidant activities of flaxseed gum films incorporated with carvacrol (The bars with the same pattern had the same carvacrol concentration and sonication power).

2,2-diphenyl-1-picrylhydrazyl (DPPH) assay, 2,2′-azinobis (3-ethylbenzothiazoline-6-sulfonic acid) (ABTS) assay, and Ferric reducing ability of plasma (FRAP) methods were used to determine the antioxidant properties of FG-C films. FG films with higher carvacrol content increased in antioxidant properties and exhibited a dose-dependent increment (*p <* 0.05, Figure 7). Similar results were also reported in bovine gelatin films incorporated with carvacrol [24], polypropylene films with carvacrol [51], and flaxseed gum-sodium alginate active films with carvacrol [52]. Compared with antioxidant capacity methods, the addition of carvacrol could improve both the reducing and radical scavenging effects of FG films. DPPH and ABTS results of FG-C films were remarkably (*p* < 0.05) affected by sonication and improved the antioxidant activities of FG-C films. The difference in the releasing rate of carvacrol could partly explain this phenomenon.

## 3. Materials and Methods 

### 3.1. Preparation of the Active Films

FG powder (1.5%, *w*/*v*) and plasticizer (glycerol, 1:3 gly/FG) were added into deionized water and stirred at 60 °C for 4 h. Carvacrol (purity of 99%, Aladdin Biochemical Technology Co., Ltd, Shanghai, China) with different mass fractions (0.05%, 0.1%, and 0.2%) was added into the film-forming solution and stirred continuously for 6 h. Then, the FG-C film-forming solution was prepared by sonication using a 20 kHz ultrasonic assisted processor (XEB-1000-P, Xiecheng ultrasonic assisted Equipment co. LTD, Shandong, China). Different sonication powers (0, 400, and 600 W/cm^2^, respectively) and sonication time (0, 15, and 30 min, respectively) were applied with a conical tip (13 mm end diameter) dip immersed 1 cm below the liquid surface. Table 1 lists the FG-C film-forming solutions treated with different sonication powers and time; the sonication was pulsed on and off for 3 s each. The FG-C film-forming solutions in the beaker were placed in cold water to prevent an increase in temperature [53]. Five hundred milliliters of film solution was poured into a horizontal glass plate (40 × 40 cm) and dried (at 25 °C, 50% relative humidity (RH)) in a constant climate chamber (KBF 240, Binder GmbH., Tuttlingen, Germany) for 36 h (the levelness of the glass plate must be guaranteed).

### 3.2. Film Thickness

A helical micrometer was used to randomly measure 12 points on the membrane surface with an accuracy of 0.001 mm (Meinaite, Shanghai, China), and the average values were taken to ensure the consistency of the results.

### 3.3. Mechanical Properties

According to the method of Ebrahimi et al. [54], the TS and EB of FG-C films were determined after 36 h treatment in 50% RH. Films were cut into rectangular strips (1.5 × 10 cm) and measured by TA. XT express (Stable Micro System, Godalming, UK). The average values of six experiments were taken for each film. The original separation distance and speed were adjusted to 50 mm and 0.8 mm·s^−1^, respectively. TS was calculated according to Equation (1):

(1) $$\mathrm{TS}=\frac{F}{A}$$

where TS is the tensile strength (Pa), F is the force (N) at maximum load, and A is the initial cross-sectional area (m^2^).

The EB (%) was calculated by dividing the extension-at-break of the specimen by the initial gauge length.

### 3.4. Contact Angle Measurements

The contact angle of the FG-C film was measured by the sessile drop method. At 25 °C, a flat diaphragm (15 × 15 mm) was fixed on the sample table of the contact angle meter (DataPhysics Instruments GmbH, Filderstadt, Germany), and 5 µL deionized water droplets were vertically and slowly applied to the diaphragm with a micro-syringe [55]. Three drops were taken from each sample for the experiment, each drop was repeated six times, and the measured data were averaged. The spreading process was monitored by a high-speed camera with a setting of 25 frames per second. The internal software of the contact angle meter was used for automatic image processing.

### 3.5. Film Opacity

The opacity of FG-C films was measured by a UV/vis spectrophotometer (UV 1800, Shimadzu Corporation, Japan). The FG-C film was cut into strips (1.0 × 4.5 cm) and placed in the interior of the dish. The opacity was measured at a wavelength of 600 nm. The empty dish was used as the illumination. Opacity is calculated according to Equation (2):

(2) $$\mathrm{Opacity}=\frac{A_{\mathrm{bs}600}}{\delta }$$

where A_bs600_ is the value of absorbance at 600 nm and δ is the thickness of film (mm).

### 3.6. WVP

*WVP* (g H_2_Om^−^^2^s^−1^MPa^−1^) of FG-C films was calculated through gravimetric analysis according to González Sandoval et al. [56] with a few modifications. Anhydrous CaCl_2_ was placed in an acrylic cup with the top of the CaCl_2_ about 5 mm from the mouth of the cup. A film with uniform thickness was selected, and three parallel samples were made for each sample, sealed in the mouth of the acrylic cup, weighed, and put into the dryer with saturated NaCl solution at the bottom. The relative humidity was 75%. During the experiment, the temperature was maintained at 20 °C and the relative humidity was 75%, with weighing once every 24 h. The water vapor transmission coefficient is calculated by the following Equation (3):

(3) $$WVP=\frac{WVTR}{A}\frac{\delta }{\Delta \mathrm{Pv}}$$

where *WVTR* is the *WVP* rate (g∙s^−1^) obtained as the slope of the linear regression of time versus weight gain; A is the area of the bottle mouth (m^2^); δ is the film thickness (*m*); and ΔPv is the vapor pressure difference between the two sides of the film. More specifically, *WVTR* is the slope obtained by plotting the final weight minus the initial weight of the sample (*W_f_*−*W_0_*) as a function of time (*t*).

### 3.7. Water Sorption Isotherms

On the basis of Slavutskyand Bertuzzi [57], a Guggenheim–Anderson–de Boer (GAB) isothermal hygroscopic model was established. FG-C films (2.0 × 2.0 cm) were kept at 50 °C for 12 h until they reached a constant weight. Then, the films were precisely weighed (±0.0001 g) into pre-weighed glass vials and balanced in desiccators containing saturated salt solutions with known water activities (LiCl-0.11, CH_3_COOK-0.22, MgCl_2_-0.33, KCO_3_-0.43, NaCl-0.75, KCl-0.84, and BaCl_2_-0.90 at 25 °C). When the film’s weight changed no more than 0.1 % every day for two consecutive times, we assumed that the film’s moisture absorption had reached equilibrium. The quality of the model fit was evaluated through the R^2^.

(4) $$W_{e}=\frac{w_{0}\times C\times k\times a_{w}}{\left(1-k\times a_{w})\times (1-k\times a_{w}+C\times k\times a_{w}\right)}$$

where *W_e_* is the equilibrium moisture content (g water/100 g dry film), *w_0_* is the monolayer content (g water/100 g dry film), and *C* and *k* are constants of the model.

### 3.8. FTIR

A Fourier transform infrared spectrometer (PerkinElmer, Fremont, California, USA) was used to detect functional groups of film samples and possible changes caused by addition of carvacrol. The film was ground into a fine powder, quickly frozen with liquid nitrogen, and pressed into sheets; the scanning range was 400–4000 cm^−1^ at a resolution of 4 cm^−1^ with 32 scans [58].

### 3.9. DSC Determination

DSC patterns of the FG-C films were analyzed by DSC Q2000 (TA Instruments, New Castle, Delaware, USA). The samples (about 13.5 mg) were sealed in aluminum pots in a nitrogen atmosphere and heated from 50 to 250 °C at 10 °C/min.

### 3.10. Antibacterial Activity

The antibacterial activities of the FG-C films were determined by the method provided by Aguilar-Sánchez et al. [59]. *S. aureus, V. parahemolyticus, S. putrefaciens,* and *P. fluorescens* were determined and the results were expressed as theinhibition ratio (%). After samples were cut into pieces, 0.5g of different film samples were added to 50mL bacterial culture medium, and then different bacterial suspension (10^6^ cells/mL) was added. The samples were cultured with shaking (150 rpm) at 30 °C for 24h. Each sample solution was uniformly coated with 100 µL of solid medium and cultured at 30 °C for 24 h. 

Inhibition ratio (%) = (colony count of FG films in the control group − the colony count of FG-C composite films in the experimental group)/colony count of FG film in the control group × 100 %.

### 3.11. Antioxidant Activity

The antioxidant activities of FG-C films were measured through ABTS, DPPH, and FRAP assays, according to Govindaswamy et al. [60], Díaz et al. [61], and Polumackanycz et al. [62], respectively. Take 1 g film samples and mix them with 10 mL of ethanol; react with DPPH, ABTS, and FRAP working solutions; and measure the UV absorbance at 517, 734, and 593 nm, respectively.

### 3.12. SEM Analysis

The microstructure of FG-C films was analyzed by SEM (MIRA3, Tescan, Czech Republic) at 5 kV accelerated voltage [63]. Before the experiment, the FG-C films were fractured in liquid nitrogen and coated with a platinum layer, and all images were displayed at a magnification of 1000×.

### 3.13. Statistical Analysis

Statistical product and service solutions (SPSS) 22.0 software was used to process the experimental data and the one-way analysis of variance (ANOVA) procedure followed by Duncan’s multiple range tests was used to determine the significant difference (*p* < 0.05) between treatments. The results were expressed as means ± SD of three independent experiments.

## 4. Conclusions

In this study, sonication was successfully applied in the production of FG-C films. Sonication at different powers and time significantly affected the mechanical, barrier, optical, physical, and microstructural properties, as well as antibacterial and antioxidant activities of FG-C films, owing to improved distribution of carvacrol. The addition of carvacrol could decrease the FG molecular internal interactions, and sonication could result in a more homogeneous dispersion of carvacrol in the FG matrix. Therefore, a higher carvacrol content in FG films resulted in less homogeneity and integrity, along with decreased mechanical and barrier properties and film opacity. In addition, the thermal properties of FG-C films with the addition of carvacrol were further supported and were consistent with the changes in molecular interactions detected by FTIR analysis and SEM observations of cross-sectional morphology. The enrichment of FG-C films ensured antibacterial properties against some spoilagemicroorganisms, as well as increased antioxidant properties, which were both enhanced by sonication.

## Figures and Tables

**Table 1:** Sample preparation with different sonication powers and time.

Number	Sample	Carvacrol Content (Mass Fraction, %)	Power (W/cm^2^)	Time (min)
1	FG-0.5C	0.05	-	-
2	FG-0.5C-4015	0.05	400	15
3	FG-0.5C-4030	0.05	400	30
4	FG-0.5C-6015	0.05	600	15
5	FG-0.5C-6030	0.05	600	30
6	FG-1.0C	0.1	-	-
7	FG-1.0C-4015	0.1	400	15
8	FG-1.0C-4030	0.1	400	30
9	FG-1.0C-6015	0.1	600	15
10	FG-1.0C-6030	0.1	600	30
11	FG-2.0C	0.2	-	-
12	FG-2.0C-4015	0.2	400	15
13	FG-2.0C-4030	0.2	400	30
14	FG-2.0C-6015	0.2	600	15
15	FG-2.0C-6030	0.2	600	30

**Table 2:** Effect of sonication onthe mechanical properties and moisture resistance of flaxseed gum films incorporated with carvacrol.

Films	Thickness (mm)	Tensile Test		WVP (g H_2_O m^−^^2^ s^−1^ MPa^−1^)
		TS (MPa)	EB (%)	
FG-0.5C	0.081 ± 0.002	25.46 ± 1.81	3.60 ± 0.09	1.90 ± 0.16
FG-0.5C-4015	0.080 ± 0.001	25.22 ± 1.98	3.79 ± 0.16	2.72 ± 0.13
FG-0.5C-4030	0.081 ± 0.001	32.29 ± 1.33	4.04 ± 0.07	1.10 ± 0.21
FG-0.5C-6015	0.083 ± 0.002	30.09 ± 1.24	4.19 ± 0.30	2.74 ± 0.33
FG-0.5C-6030	0.083 ± 0.002	33.40 ± 1.68	4.46 ± 0.40	1.57 ± 0.11
FG-1.0C	0.084 ± 0.001	22.81 ± 0.93	2.69 ± 0.28	2.31 ± 0.21
FG-1.0C-4015	0.085 ± 0.001	23.60 ± 1.50	3.22 ± 0.33	2.46 ± 0.11
FG-1.0C-4030	0.081 ± 0.002	24.32 ± 1.40	3.30 ± 0.05	1.80 ± 0.25
FG-1.0C-6015	0.081 ± 0.001	27.04 ± 1.79	3.54 ± 0.05	2.27 ± 0.29
FG-1.0C-6030	0.081 ± 0.001	29.16 ± 0.85	3.84 ± 0.19	1.99 ± 0.22
FG-2.0C	0.085 ± 0.002	18.45 ± 1.22	2.60 ± 0.23	2.51 ± 0.39
FG-2.0C-4015	0.085 ± 0.002	21.81 ± 1.13	2.66 ± 0.20	1.79 ± 0.33
FG-2.0C-4030	0.082 ± 0.002	23.08 ± 1.50	3.02 ± 0.21	2.07 ± 0.19
FG-2.0C-6015	0.084 ± 0.001	25.32 ± 0.92	2.88 ± 0.05	1.31 ± 0.14
FG-2.0C-6030	0.083 ± 0.002	27.08 ± 1.10	3.06 ± 0.18	1.67 ± 0.29

TS—tensile strength; EB—elongation at break; WVP—water vapor permeability.

**Table 3:** Guggenheim–Anderson–de Boer (GAB) isothermal moisture absorption model parameters and fitting accuracy of flaxseed gum films incorporated with carvacrol.

Films	*W_0_* (%)	*C*	*k*	*R^2^*
FG-0.5C	7.2524	15.0117	0.0099	0.9894
FG-0.5C-4015	9.9610	4.0095	0.0098	0.9939
FG-0.5C-4030	7.9505	5.9960	0.0100	0.9831
FG-0.5C-6015	10.2233	1.9750	0.0097	0.9918
FG-0.5C-6030	10.3772	3.3244	0.0096	0.9948
FG-1.0C	7.0905	27.2029	0.0100	0.9954
FG-1.0C-4015	10.4380	5.2432	0.0098	0.9986
FG-1.0C-4030	13.2619	1.4699	0.0094	0.9994
FG-1.0C-6015	11.2298	3.0018	0.0095	0.9951
FG-1.0C-6030	8.5154	8.8591	0.0100	0.9826
FG-2.0C	−11.8895	1.7437	−0.0126	0.9989
FG-2.0C-4015	9.1218	5.4526	0.0097	0.9945
FG-2.0C-4030	8.7280	5.8071	0.0098	0.9936
FG-2.0C-6015	−8.1933	1.2650	−0.037	0.9856
FG-2.0C-6030	12.4246	1.8996	0.0094	0.9978

*W_0_*—monolayer content (g water/100 g dry film); *C*—constant of the model; *k*—constants of the model; R^2^—goodness of fit.

**Table 4:** Effect of sonication on the opacity of flaxseed gum films incorporated with carvacrol.

Films	Thickness(mm)	Opacity(mm^−1^)
FG-0.5C	0.081 ± 0.002	0.925 ± 0.007
FG-0.5C-4015	0.080 ± 0.001	0.981 ± 0.008
FG-0.5C-4030	0.081 ± 0.001	0.975 ± 0.020
FG-0.5C-6015	0.083 ± 0.002	0.946 ± 0.005
FG-0.5C-6030	0.083 ± 0.002	0.953 ± 0.005
FG-1.0C	0.084 ± 0.001	0.866 ± 0.013
FG-1.0C-4015	0.085 ± 0.001	0.879 ± 0.017
FG-1.0C-4030	0.081 ± 0.002	0.929 ± 0.009
FG-1.0C-6015	0.081 ± 0.001	0.922 ± 0.019
FG-1.0C-6030	0.081 ± 0.001	0.933 ± 0.017
FG-2.0C	0.085 ± 0.002	0.821 ± 0.010
FG-2.0C-4015	0.085 ± 0.002	0.852 ± 0.018
FG-2.0C-4030	0.082 ± 0.002	0.884 ± 0.010
FG-2.0C-6015	0.084 ± 0.001	0.866 ± 0.019
FG-2.0C-6030	0.083 ± 0.002	0.880 ± 0.012
